# Low Digital Engagement, Depressive Symptoms, and Probable Cognitive Impairment Among Community-Dwelling Older Adults in Shanghai: A Cross-Sectional Path Analysis of Self-Perceived Burden, Uselessness, and Loneliness

**DOI:** 10.3390/bs16101726

**Published:** 2026-09-23

**Authors:** Renhao Li, Xiaomin Qu, Fangzhou Ye, Ting Xu, Xichenhui Qiu, Katherine Wang, Wanli Zang, Zhongfang Yang, Zheng Zhu, Xiang Qi

**Affiliations:** 1 Rory Meyers College of Nursing, New York University, New York, NY 10010, USA; rl5235@nyu.edu; 2 School of Social Development, East China University of Political Science and Law, Shanghai 201620, China; 3 School of Global Public Health, New York University, New York, NY 10003, USA; 4 School of Nursing, Nanjing Medical University, Nanjing 211166, China; 5 Health Science Center, Shenzhen University, Shenzhen 518060, China; 6 Albert Einstein College of Medicine, Bronx, New York, NY 10461, USA; 7 School of Physical Education, Soochow University, Suzhou 215006, China; 8 School of Nursing, Soochow University, Suzhou 215005, China; 9 School of Nursing, Fudan University, Shanghai 200433, China

**Keywords:** low digital engagement, loneliness, self-perceived burden, self-perceived uselessness, cognitive impairment, depressive symptoms, older adults

## Abstract

Low engagement with digital technologies is increasingly recognized as a health-related correlate in later life, yet the psychosocial variables linking it to mental and cognitive health remain unclear. Guided by the stress-process model, this cross-sectional study examined whether the associations of low digital engagement (LDE) with depressive symptoms and probable cognitive impairment were consistent with indirect pathways through self-perceived burden, self-perceived uselessness, and loneliness. We analyzed data from 1703 community-dwelling adults aged 55 to 85 years in Shanghai, China, collected between 2022 and 2023. Two path models specified self-perceived burden and self-perceived uselessness as parallel first-stage mediators and loneliness as a second-stage mediator: a linear model for depressive symptoms (CES-D-10) and a logit model for probable cognitive impairment (AD8 ≥ 2), each adjusting for sociodemographic, lifestyle, and health covariates (5000 bootstrap replications). Greater LDE was associated with more depressive symptoms (b = 0.062, 95% CI: 0.011, 0.113) and higher odds of probable cognitive impairment (OR = 1.040 per point, 95% CI: 1.014, 1.067). For depressive symptoms, the total indirect association accounted for 75.2% of the total association: significant specific indirect components included burden (30.3%), uselessness (15.6%), and the serial chains via burden and loneliness (8.7%) and via uselessness and loneliness (7.0%). The direct association was not statistically significant. For probable cognitive impairment, the direct association remained significant, and the significant indirect components were limited to the burden route. Formal contrasts indicated no significant difference between the burden and uselessness routes. The association between LDE and loneliness was attenuated to non-significance once the self-evaluative variables were included. Reverse models fit the data equally well. These cross-sectional associations generate hypotheses for future longitudinal and intervention research in rapidly digitizing urban settings.

## 1. Introduction

China is experiencing one of the world’s most rapid population aging transitions (WHO, n.d.). By 2024, more than one-fifth of the national population was aged 60 years or older, and in large cities such as Shanghai the proportion exceeded one third of residents (Chu & Lee, 2026; Xinhua, 2024). Late-life depression and cognitive impairment are common: in 2024, 13.8% of Chinese adults aged 60 years or older had depression, and in 2020, 15.5% had mild cognitive impairment (Jia et al., 2020; Gao et al., 2025). As longevity increases, protecting mental and cognitive health in later life has become an urgent public health priority. Identifying modifiable behavioral and social factors associated with these outcomes is therefore essential for prevention.

Digital information and communication technologies (ICTs) may support healthy aging by expanding access to information, services, and social connection (Qi et al., 2025; Qi & Wu, 2024). Researchers increasingly recognize digital engagement as a continuum that ranges from non-use to initial adoption and sustained use. Within this continuum, some older adults may gradually reduce their participation in digital activities or use technologies less frequently over time (Olphert & Damodaran, 2013). It has been consistently associated with adverse health outcomes among older adults, including functional limitations, cognitive impairment, depressive symptoms, and social isolation, (X. Du et al., 2023; S. Li et al., 2025). Recent research has highlighted a significant association between low digital engagement (LDE) and depressive symptoms and cognitive impairment in older adults (B. Chen et al., 2024; X. Du et al., 2023), but the pathways underlying these associations remain poorly understood. In the present study, we use “low digital engagement” to encompass both non-use and low-intensity use of digital technology.

As digital technologies become central to social interaction, health care access, financial transactions, transportation, and community participation, older adults who engage less with digital tools may face practical barriers that extend into social life (X. Du et al., 2023; Mubarak & Suomi, 2022; Seifert et al., 2021; Wang et al., 2025). Limited digital engagement may increase dependence on family members for everyday tasks, including medical appointments, online payments, and transportation applications. This dependence may affect how older adults evaluate their own role in the family. They may begin to feel burdensome when they require repeated help, or useless when they are unable to contribute in digitally mediated settings. These self-evaluations provide a plausible bridge between low digital engagement and loneliness. Research on technology acceptance further suggests that perceived ease of use and perceived usefulness shape older adults’ adoption and continued use of digital tools (K. Chen & Chan, 2014; Davis, 1989), and that concerns about online security, privacy, and fraud, together with complex interfaces, are prominent deterrents to digital engagement in later life (Mubarak & Suomi, 2022).

Self-perceived burden, uselessness, and loneliness are important psychological risk factors in later life. Self-perceived burden refers to the belief that one’s needs impose an unfair load on others (Cukrowicz et al., 2011), whereas self-perceived uselessness reflects doubts about one’s value to family and society. Older adults who frequently feel burdensome or useless are more likely to report depressive symptoms and cognitive impairment (Gu et al., 2016). These self-perceptions may also contribute to loneliness. When older adults believe they impose on others or have little value to offer, they may withdraw from social interaction, avoid asking for support, or feel less entitled to belonging (Q. Du et al., 2022; Gu et al., 2016). Loneliness, in turn, is consistently associated with depressive symptoms and poorer cognitive outcomes (Dong et al., 2025; Qi et al., 2023). This reasoning suggests two serial pathways from low digital engagement to mental and cognitive health: one through self-perceived burden and loneliness, and the other through self-perceived uselessness and loneliness (Gu et al., 2016; Zhao et al., 2017).

From the stress-process perspective, mental health outcomes arise from sources of stress that operate through psychosocial mediators to produce psychological manifestations (Pearlin et al., 1981). Within this framework, low digital engagement can be a chronic strain of later life. As essential services and social interactions increasingly move online, older adults with lower levels of digital engagement may encounter recurring practical and social challenges. The Stress Process Model further emphasizes that the associations between such strains and health outcomes are often conditioned by self-evaluations and social resources. Accordingly, this framework provides a theoretical basis for examining whether the association between low digital engagement and mental or cognitive outcomes is consistent with indirect pathways through self-perceived burden or uselessness and, subsequently, loneliness.

Previous studies have examined internet use in relation to depression and cognition using mediation models (Y. Du et al., 2026; X. Li et al., 2025; Zhang et al., 2025), but no study has jointly tested whether self-perceived burden and uselessness operate sequentially with loneliness as serial mediators. Based on these considerations, we tested four linked hypotheses: (1) low digital engagement would show a significant total association with depressive symptoms and probable cognitive impairment (total association, c); (2) low digital engagement would have a specific indirect association with outcomes through the first-stage mediators, self-perceived burden and self-perceived uselessness (indirect association a_1_ × b_1_, a2 × b_2_); (3) low digital engagement would have a specific indirect association on outcomes through loneliness (indirect association a_3_ × b_3_); (4) low digital engagement would have a serial indirect association with outcomes through the first-stage mediators and loneliness (a_1_ × d_1_ × b_3_ + a2 × d_2_ × b_3_).

The conceptual framework is presented in Figure 1.

## 2. Methods

### Study Design and Sample

This study used cross-sectional data from a population-based survey of community-dwelling older adults conducted in Shanghai, China, between November 2022 and April 2023. A multistage sampling design was used to obtain a geographically diverse sample of residents aged 55 to 85 years. The lower bound of 55 years follows common practice in Chinese aging research and aligns with the statutory retirement age for many urban women. We use the term “older adults” for brevity, while noting that participants aged 55 to 59 years do not meet a conventional ≥60-year definition of older adult. Sampling proceeded through four hierarchical stages. First, 10 districts were randomly selected from all districts in Shanghai. Second, two streets or towns were randomly selected within each sampled district, yielding 20 streets or towns. Third, two residential or village committees were randomly selected within each sampled street or town, producing 40 primary sampling units. Finally, more than 40 eligible older adults were recruited within each residential or village committee through quota sampling, balanced by sex and age distribution in Shanghai (Ma et al., 2025). One eligible older adult was randomly selected from each approached household. A total of 1707 participants completed the survey out of approximately 2100 households approached. The numbers of ineligible households and individual refusals were not recorded separately. Post-stratification weights were calculated using Shanghai’s age-sex distribution, yielding age-sex post-stratified descriptive estimates for the city’s approximately 6.49 million adults aged 55–85 years. After excluding four participants with incomplete data on key variables, the final analytic sample comprised 1703 participants with complete data on the exposure, mediators, outcomes, and covariates.

## 3. Measures

### 3.1. Low Digital Engagement

Low digital engagement was operationalized as an index combining lack of access to and lower intensity of digital technology use (Y. Du et al., 2026). Participants reported how often they used each of five digital technologies, including smartphone, wearable device, computer, tablet, and the internet, on a 4-point scale from 1 (frequently) to 4 (never); participants without access to a given technology were assigned a score of 5 for that item. Responses were summed to create a total score (range: 5 to 25), with higher scores indicating lower digital engagement. Comparable indices have been used to capture lower digital engagement among older adults, including in Chinese community samples, and have demonstrated good internal consistency and associations with health outcomes (Y. Du et al., 2026). The present measure captures both access to and frequency of use of digital technologies and has been shown to predict adverse health outcomes in large-scale longitudinal studies, including functional dependence across five international cohorts and depressive symptoms across 24 countries (S. Li et al., 2025; Lu et al., 2022; Wang et al., 2025). We acknowledge that this approach does not capture digital skills or quality of use, which may operate through distinct pathways and should be examined in future research.

### 3.2. Mediators

Three psychosocial variables were examined as potential mediators between lower digital engagement and health outcomes. Self-perceived burden was measured using a 14-item scale assessing perceptions of being a burden to others and to society (Cousineau et al., 2003). Items captured emotional, financial, social, and caregiving concerns, such as ‘I worry that taking care of me places too much financial burden on my family.’ Participants indicated how often they experienced each feeling during the past month on a 5-point Likert scale from 1 (never) to 5 (always). Items reflecting positive contributions to family or society were reverse-coded so that higher scores consistently indicated greater perceived burden. Items were summed to generate a total self-perceived burden score (range: 14 to 70), with Cronbach’s alpha of 0.81 in this sample. Self-perceived uselessness was measured using an 8-item scale capturing concerns about loss of social value, personal significance, and usefulness in later life, such as ‘After getting old, my life will lose its meaning.’ (Zhao et al., 2017). Each item was rated on a 4-point scale from 1 (not worried at all) to 4 (very worried). Item scores were summed to produce a total self-perceived uselessness score (range: 8 to 32), with higher values indicating stronger feelings of uselessness and diminished self-worth. Cronbach’s alpha was 0.87. Loneliness was assessed using the three-item UCLA Loneliness Scale (Hughes et al., 2004). Items included ‘I feel left out,’ ‘I feel isolated from others,’ and ‘I feel that I lack companionship.’ Response options ranged from 1 (never) to 4 (always). Scores were summed to create a total loneliness score (range: 3 to 12), with higher scores indicating greater loneliness. Cronbach’s alpha was 0.88.

### 3.3. Outcomes

We examined two outcome domains. Depressive symptoms were measured by the 10-item Center for Epidemiological Studies Depression scale (CES-D-10) (Irwin et al., 1999). Total scores ranged from 0 to 30, with higher scores indicating greater depressive symptomatology. CES-D-10 scores were modeled as a continuous outcome. Probable cognitive impairment was evaluated using the AD8 Dementia Screening Interview (Galvin et al., 2005), an eight-item yes or no screening measure scored from 0 to 8. Consistent with the established screening threshold, participants with AD8 scores of 2 or higher were classified as having probable cognitive impairment, whereas those with scores below 2 were classified as screen-negative for probable cognitive impairment. The binary outcome was modeled using a logit link. Because the AD8 is a brief self-reported screening instrument, AD8 ≥ 2 indicates probable rather than clinically confirmed cognitive impairment.

### 3.4. Covariates

Models adjusted for demographic, socioeconomic, lifestyle, and health-related covariates. Demographic covariates included age, sex (male or female), marital status (married or cohabiting versus not partnered), hukou status (urban versus rural household registration, used as a proxy for lifelong urban or rural residency and associated social entitlements), education level (less than high school, high school, and more than high school), and annual household income quartile. Health-related covariates included body mass index (BMI), categorized according to Chinese guideline cutoffs (Tang et al., 2025), as underweight (<18.5 kg/m^2^), normal weight (18.5 to 23.9 kg/m^2^), overweight (24.0 to 27.9 kg/m^2^), or obese (≥28.0 kg/m^2^), current smoking status, frequent alcohol consumption. Health-related covariates also included the number of self-reported chronic conditions (hypertension, diabetes, heart disease, stroke or cerebrovascular disease, bronchitis or lung disease, arthritis, and cancer; range 0–7), any limitation in basic activities of daily living (Barthel Index < 100), living alone, and the number of relatives and friends in regular contact.

### 3.5. Statistical Analyses

Descriptive statistics were calculated for all study variables. Continuous variables were summarized using means and standard deviations, and categorical variables were summarized using frequencies and percentages. Zero-order Pearson correlations were computed to examine bivariate associations among core study variables. To examine associations between lower digital engagement and health outcomes, as well as potential indirect association through psychosocial factors, we used path analysis within a structural equation modeling framework. Separate mediation models were specified for depressive symptoms and cognitive impairment.

To evaluate the hypothesized mediation pathways, we first fitted single-mediator models for each psychosocial variable: self-perceived burden, self-perceived uselessness, and loneliness. To examine whether the associations between low digital engagement and the two outcomes were consistent with the hypothesized indirect pathways, we estimated two separate path models within a structural equation modeling framework. In each model, self-perceived burden and self-perceived uselessness were specified as parallel first-stage mediators with correlated residuals, loneliness as a second-stage mediator. Depressive symptoms and probable cognitive impairment were modeled separately as the outcome in separate analyses. All equations were adjusted for the full set of covariates.

For each outcome, the total association was decomposed into a direct component and five specific indirect components: through burden only, through uselessness only, through loneliness only, and through the two serial chains (burden → loneliness and uselessness → loneliness). Standard errors were obtained by nonparametric bootstrap with 5000 replications; confidence intervals for products and contrasts of coefficients are delta-method, normal-based intervals computed from the bootstrap covariance matrix. Because all variables were observed and all hypothesized paths were freely estimated, the primary structural model was just identified (df = 0) and global fit indices are not defined; we therefore additionally estimated an over-identified model fixing the non-significant direct path from low digital engagement to loneliness to zero and report its fit indices together with a likelihood-ratio test against the saturated model. Because this constraint was chosen based on the non-significant path in the primary model, this comparison serves as a parsimonious sensitivity analysis rather than a test of the hypothesized structure. The path models were estimated without sampling weights because all models adjust for the variables used to construct the post-stratification weights (age and sex), in which case unweighted estimates are consistent and more precise (Solon et al., 2015; Winship & Radbill, 1994) and the weights were used for the age-sex post-stratified descriptive estimates in Table 1. The main path models used 5000 bootstrap replications; single-mediator and sensitivity analyses used 1000 replications. Analyses were conducted using Stata 18.0.

## 4. Results

### 4.1. Sample Characteristics

Table 1 presents the descriptive characteristics of the analytic sample (*N* = 1703), weighted and unweighted. The mean age was 68.59 years (SD = 6.65), and 58.4% of participants were female. Most participants were married or cohabiting (83.0%) and held an urban hukou (70.0%). More than half had less than a high school education (51.5%). Participants reported a mean of 1.44 chronic conditions (SD = 1.25); 10.0% had a limitation in basic activities of daily living, 13.5% lived alone, and participants maintained regular contact with a mean of 20.1 relatives and friends. Household income was distributed across quartiles, with 25.0% in the lowest quartile and 21.2% in the highest quartile. Most participants were of normal weight (48.6%) or overweight (36.5%); 18.9% were current smokers and 11.1% were frequent drinkers. The mean low digital engagement score was 16.07 (SD = 4.78; possible range: 5 to 25). Among the psychosocial mediators, the mean self-perceived burden score was 29.16 (SD = 9.03; possible range: 14 to 70), the mean self-perceived uselessness score was 15.57 (SD = 5.49; possible range: 8 to 32), and the mean loneliness score was 3.72 (SD = 1.71; possible range: 3 to 12). The mean depressive symptom score (CES-D-10) was 5.56 (SD = 4.64; possible range: 0 to 30), and 727 participants (42.7%) screened positive for probable cognitive impairment (AD8 ≥ 2). After post-stratification weighting on age and sex, estimates are aligned with the age-sex structure of approximately 6.49 million Shanghai residents aged 55 to 85 years; relative to the unweighted sample, the weighted population was younger (mean age 66.11 years), included a higher proportion of men (49.1%), and had a lower prevalence of probable cognitive impairment (39.5%).

Supplementary Table S1 presents the means, standard deviations, and Pearson correlations among the key study variables. Low digital engagement was significantly and positively correlated with self-perceived burden (r = 0.221, *p* < 0.001), self-perceived uselessness (r = 0.097, *p* < 0.001), depressive symptoms (r = 0.159, *p* < 0.001), and AD8 scores (r = 0.236, *p* < 0.001). Self-perceived burden was moderately correlated with self-perceived uselessness (r = 0.399, *p* < 0.001), suggesting that these constructs are related but distinct. Both self-perceived burden and self-perceived uselessness were substantially correlated with depressive symptoms (r = 0.437 and r = 0.402, respectively) and AD8 scores (r = 0.285 and r = 0.249, respectively). Loneliness was positively correlated with low digital engagement (r = 0.124), self-perceived burden (r = 0.294), self-perceived uselessness (r = 0.295), depressive symptoms (r = 0.498), and AD8 scores (r = 0.285; all *p* < 0.001).

### 4.2. Associations of Low Digital Engagement with Mediators and Outcomes

Supplementary Table S2 presents the multivariable linear regression results for associations between lower digital engagement and each mediator and outcome. After adjustment for the full covariate set, Lower digital engagement was significantly associated with higher self-perceived burden (b = 0.187, *p* < 0.001) and higher self-perceived uselessness (b = 0.069, *p* = 0.034); the association with loneliness was positive but borderline (b = 0.019, *p* = 0.056). Lower digital engagement was also associated with more depressive symptoms (b = 0.062, *p* = 0.017) and with higher odds of probable cognitive impairment (OR = 1.040 per point, *p* = 0.003). Coefficients in this section are unstandardized (odds ratios for the binary outcome).

Notable gradients were observed among the covariates. Being partnered was associated with lower loneliness (b = −0.974, *p* < 0.001) and fewer depressive symptoms (b = −1.030, *p* < 0.01). Higher household income was associated with lower self-perceived burden (b = −1.273 per quartile, *p* < 0.001), lower self-perceived uselessness (b = −0.339, *p* < 0.05), and fewer depressive symptoms (b = −0.317, *p* < 0.01). Urban hukou was associated with lower odds of probable cognitive impairment (OR = 0.642, *p* < 0.001). The number of chronic conditions and any limitation in activities of daily living were associated with all three mediators and both outcomes, and a larger number of relatives and friends in regular contact was associated with lower burden, loneliness, depressive symptoms, and odds of probable cognitive impairment.

### 4.3. Single Mediation Models

Supplementary Table S3 presents indirect associations from the single-mediator models. Low digital engagement showed significant indirect associations with depressive symptoms through self-perceived burden (b = 0.034, 95% CI: 0.016, 0.052) and through self-perceived uselessness (b = 0.020, 95% CI: 0.002, 0.038), and with probable cognitive impairment through burden (b = 0.0070 log-odds, 95% CI: 0.0028, 0.0112) and through uselessness (b = 0.0037, 95% CI: 0.0001, 0.0072). The indirect association through loneliness alone did not reach statistical significance for either outcome (depressive symptoms: b = 0.024, 95% CI: −0.003, 0.050; probable cognitive impairment: b = 0.0052, 95% CI: −0.0009, 0.0113).

### 4.4. Path Models

Figure 2 shows the depressive-symptoms path model, with path coefficients in Table 2 and the decomposition of total associations in Table 3. Greater low digital engagement was associated with higher burden (b = 0.187, 95% CI: 0.093–0.281; β = 0.099) and uselessness (b = 0.069, 95% CI: 0.007–0.131; β = 0.060). Both burden (b = 0.030, β = 0.159) and uselessness (b = 0.065, β = 0.210) were associated with loneliness (both *p* < 0.001), whereas the direct association between low digital engagement and loneliness was not significant (b = 0.009, 95% CI: −0.011 to 0.029; *p* = 0.395). Burden (b = 0.101, β = 0.196), uselessness (b = 0.141, β = 0.167), and loneliness (b = 0.963, β = 0.355) were each associated with depressive symptoms (all *p* < 0.001).

The total association between low digital engagement and depressive symptoms (b = 0.062, 95% CI: 0.011–0.113) was largely indirect (b = 0.047, 95% CI: 0.018–0.075; 75.2%); the direct association was not significant after including the mediators (b = 0.015, 95% CI: −0.027 to 0.058). Significant indirect associations were observed through burden alone (b = 0.019, 30.3%), uselessness alone (b = 0.010, 15.6%), burden → loneliness (b = 0.005, 8.7%), and uselessness → loneliness (b = 0.004, 7.0%); the loneliness-only pathway was not significant. The model constraining the direct path from low digital engagement to loneliness to zero fit well (χ^2^(1) = 0.87, *p* = 0.352; RMSEA < 0.001; CFI = 1.000; TLI = 1.005; SRMR = 0.001), with no evidence of worse fit than the saturated model (Supplementary Table S5).

Figure 3 depicts the corresponding logit model for probable cognitive impairment (AD8 ≥ 2). In the outcome equation, burden (b = 0.023 log-odds, OR = 1.023, 95% CI for b: 0.009–0.037), uselessness (b = 0.023, OR = 1.023, 95% CI: 0.001–0.046), and loneliness (b = 0.228, OR = 1.256, 95% CI: 0.152–0.304) were associated with higher odds of probable cognitive impairment. Low digital engagement also retained a significant direct association (b = 0.032, OR = 1.032, 95% CI: 0.004–0.059; *p* = 0.023). Because the outcome equation uses the log-odds scale, indirect and direct components do not sum to a total association; accordingly, proportions of the total are not reported. Constraining the direct path from low digital engagement to loneliness to zero was not rejected (LR χ^2^(1) = 0.87, *p* = 0.352), and the constrained model had lower AIC and BIC. Global fit indices are not defined for the logit model (Supplementary Table S5).

Formal contrast tests revealed no statistically significant differences in the magnitude of the burden- and uselessness-mediated pathways for either outcome. Likewise, the two serial indirect pathways did not differ significantly. Overall, these findings indicate that the two first-stage mediating pathways were comparable in magnitude, with no evidence that either pathway predominated.

Across both models, the direct association between low digital engagement and loneliness was not statistically significant after adjustment for the self-evaluative variables. The covariate-adjusted association estimated without the mediators was modest and marginally non-significant (b = 0.019, *p* = 0.056), and was further attenuated after inclusion of self-perceived burden and uselessness (b = 0.009, *p* = 0.395).

### 4.5. Sensitivity Analyses

In models placing loneliness before the self-evaluative variables, none of the re-ordered serial chains was statistically significant for either outcome. Reverse models treating each outcome as the predictor and low digital engagement as the final dependent variable yielded significant associations: the reverse chain from depressive symptoms through loneliness and burden to low digital engagement was statistically significant (b = 0.003, 95% CI: 0.0005, 0.006), and probable cognitive impairment showed both a significant direct association with low digital engagement (b = 0.497, 95% CI: 0.087, 0.907) and a significant reverse chain through loneliness and burden (b = 0.025, 95% CI: 0.007, 0.044). Reverse directionality is therefore an empirically viable alternative, and the specified ordering rests on theory rather than on the data (Supplementary Table S4).

## 5. Discussion

This cross-sectional study examined whether the associations of low digital engagement with depressive symptoms and probable cognitive impairment were consistent with indirect pathways through self-perceived burden, self-perceived uselessness, and loneliness. In a sample of 1703 community-dwelling older adults in Shanghai, low digital engagement was significantly associated with both outcomes. For depressive symptoms, 75.2% of the total association was accounted for by indirect components through the self-evaluative variables and loneliness, and the direct association was not statistically significant once these variables were included. For probable cognitive impairment, the direct association remained significant, and the significant indirect components were limited to the burden route. Formal contrasts indicated that the burden and uselessness routes did not differ significantly. Because all variables were measured concurrently, the findings represent statistical associations that are consistent with the hypothesized pathways.

Self-perceived burden showed the most consistent pattern of associations. In highly digitized urban settings, limited digital engagement may heighten dependence on family members for everyday tasks such as making medical appointments, managing finances, or using transportation applications. This dependence can erode older adults’ sense of autonomy and make them feel burdensome to others (Zhu et al., 2026). Feeling burdensome may in turn be accompanied by social withdrawal and loneliness, a pattern consistent with the interpersonal theory of suicide, which identifies perceived burdensomeness as a salient interpersonal risk state in later life (Cukrowicz et al., 2011).

Self-perceived uselessness was also associated with low digital engagement, and the formal contrast indicated that the burden and uselessness routes did not differ significantly in magnitude. Uselessness was strongly associated with loneliness (β = 0.210) and with both outcomes. Feelings of diminished social value may reflect internalized age-related stereotypes and perceived loss of role identity when older adults cannot participate in digitally mediated activities (Gu et al., 2016; Zhao et al., 2017). For probable cognitive impairment, neither the uselessness-only nor the serial uselessness–loneliness component reached statistical significance. Accordingly, the uselessness pathway should be interpreted cautiously, particularly for the cognitive outcome. More broadly, the AD8 outcome throughout reflects self-reported screening status rather than clinically confirmed cognitive impairment.

Across models, loneliness was significantly associated with both outcomes (β = 0.355 for depressive symptoms; OR = 1.256 per point for probable cognitive impairment), and the self-evaluative variables were associated with the outcomes partly through loneliness. The association between low digital engagement and loneliness was modest and borderline significant in the covariate-adjusted model without mediators (b = 0.019, *p* = 0.056), and was further attenuated after self-perceived burden and uselessness were included (b = 0.009, *p* = 0.395). This implies that association between low digital engagement and loneliness appears to overlap substantially with these self-evaluative variables. This may help explain inconsistent findings on digital technology use and loneliness: some studies report that internet use is associated with lower loneliness (Cotten et al., 2013; Qi et al., 2025), whereas others have found no significant association (Kung & Steptoe, 2023; Stockwell et al., 2021), and a meta-analysis of digital technology interventions found no evidence that such interventions reduce loneliness (Shah et al., 2021). When self-evaluative processes are not modeled, the association between digital engagement and loneliness may therefore appear weak or inconsistent. This interpretation is consistent with meta-analytic evidence linking loneliness to depressive symptoms among older adults (Wen et al., 2024) and longitudinal evidence linking loneliness with accelerated cognitive decline and dementia risk (Lara et al., 2019; Luchetti et al., 2024), although the present design cannot establish directionality.

From a stress-process perspective(Pearlin et al., 1981), sustained low digital engagement may be viewed as a structural stressor, associated with psychosocial vulnerability through two related pathways. The burden pathway is consistent with dependence-related strain arising from reliance on others for digitally mediated tasks, whereas the uselessness pathway is consistent with role-loss strain and reduced perceived social value. These findings support a dual-pathway framework that links digital inequality with the stress-process model of aging. These interpretations are conditional on the assumed ordering and require longitudinal testing.

This study has several strengths and limitations. Strengths include a community-based sample drawn through a multistage design with random selection of geographic units across Shanghai, joint modeling of two related self-evaluative variables with correlated residuals, bootstrap inference with 5000 replications, and extensive sensitivity analyses including alternative orderings and reverse models. Several limitations warrant consideration. First, the cross-sectional design precludes causal inference. Reverse causation is possible because depressive symptoms or probable cognitive impairment may reduce older adults’ motivation or capacity to use digital technologies. Reverse models in which depressive symptoms or probable cognitive impairment predict low digital engagement fit the data equally well, and several reverse associations were statistically significant. Second, self-reported measures may be subject to social desirability and recall biases. In particular, the AD8 is a brief self-reported screening instrument, and AD8 ≥ 2 indicates probable rather than clinically confirmed cognitive impairment. Third, the final sampling stage used quota sampling, and the sample is limited to Shanghai, one of China’s most digitalized cities; findings should not be generalized to older adults in rural or less digitalized settings. Fourth, the measure of low digital engagement focused on access to and frequency of use of digital technologies and did not capture digital skills, quality of use, or specific content of digital engagement, nor the reasons for low use, such as usability difficulties or security concerns. Fifth, although the models adjusted for chronic conditions, ADL limitation, living arrangement, and social contacts, residual confounding by unmeasured factors such as sensory impairment, physical activity, social support quality, and baseline technology access remains possible.

In terms of implications, these cross-sectional findings generate hypotheses rather than intervention recommendations. If the observed associations reflect processes running from low digital engagement to negative self-evaluations and loneliness, then programs that pair digital literacy training with attention to older adults’ autonomy and sense of contribution would be reasonable candidates for development and formal evaluation. Whether such programs can reduce perceived burdensomeness, loneliness, depressive symptoms, or cognitive concerns is an empirical question for future longitudinal and intervention studies.

## 6. Conclusions

This study shows that low digital engagement is associated with depressive symptoms and probable cognitive impairment among community-dwelling older adults in Shanghai. The association with depressive symptoms was predominantly indirect, through self-perceived burden, self-perceived uselessness, and loneliness, whereas the association with probable cognitive impairment was mainly direct, with a significant indirect component through the burden route. The two self-evaluative routes were comparable in magnitude. These findings are consistent with, but do not establish, psychosocial pathways linking low digital engagement to late-life mental and cognitive health. Longitudinal and intervention research is needed to determine directionality and to test whether supporting digital autonomy and addressing negative self-evaluations can benefit older adults in rapidly digitizing urban settings.

## Figures and Tables

**Figure 1:**
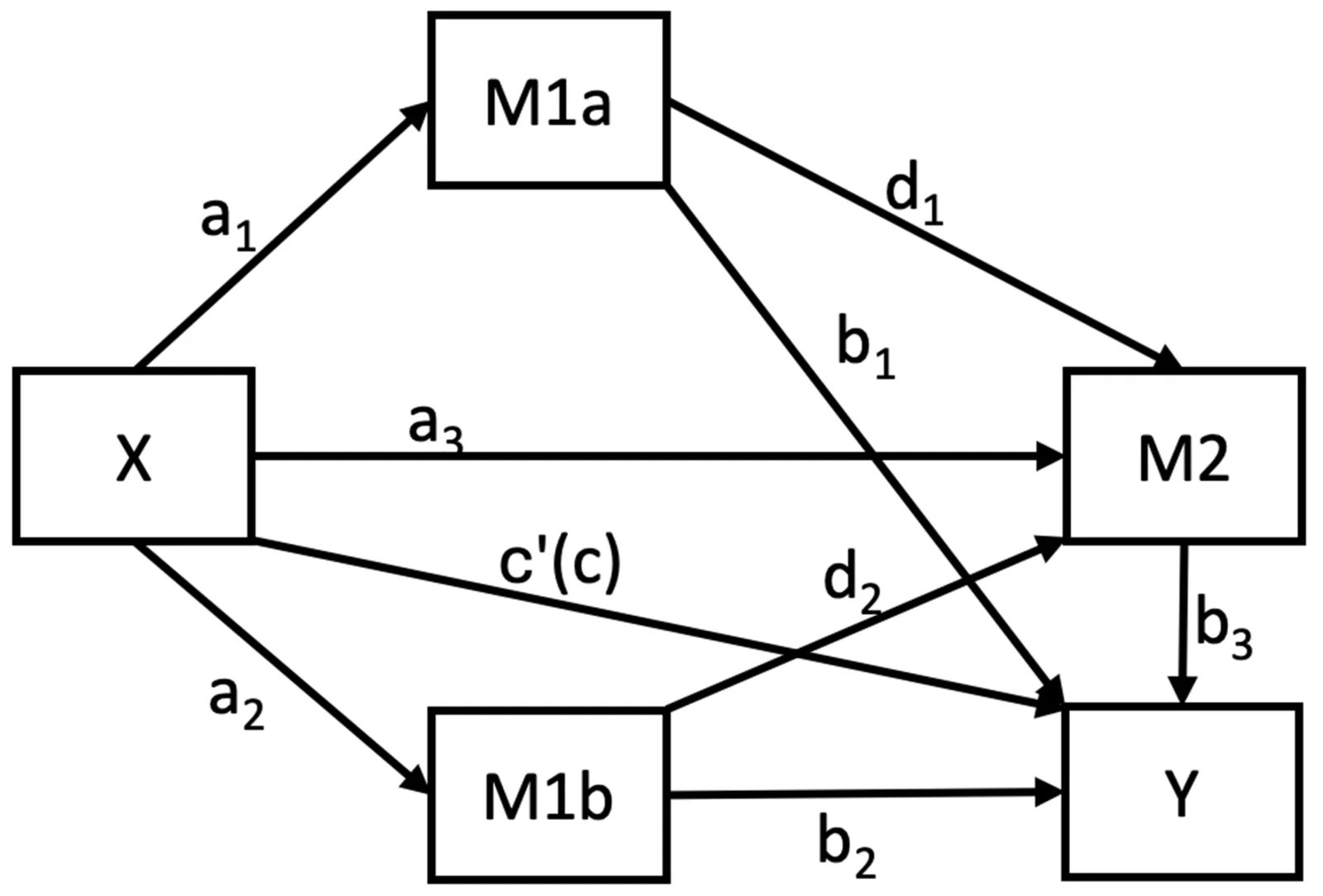
A hypothesized serial multiple mediation model. X denotes low digital engagement; M1a (self-perceived burden) and M1b (self-perceived uselessness) are parallel first-stage mediators with correlated residuals; M2 (loneliness) is the second-stage mediator; Y denotes depressive symptoms or cognitive impairment. a, b, c′, and d denote path coefficients.

**Figure 2:**
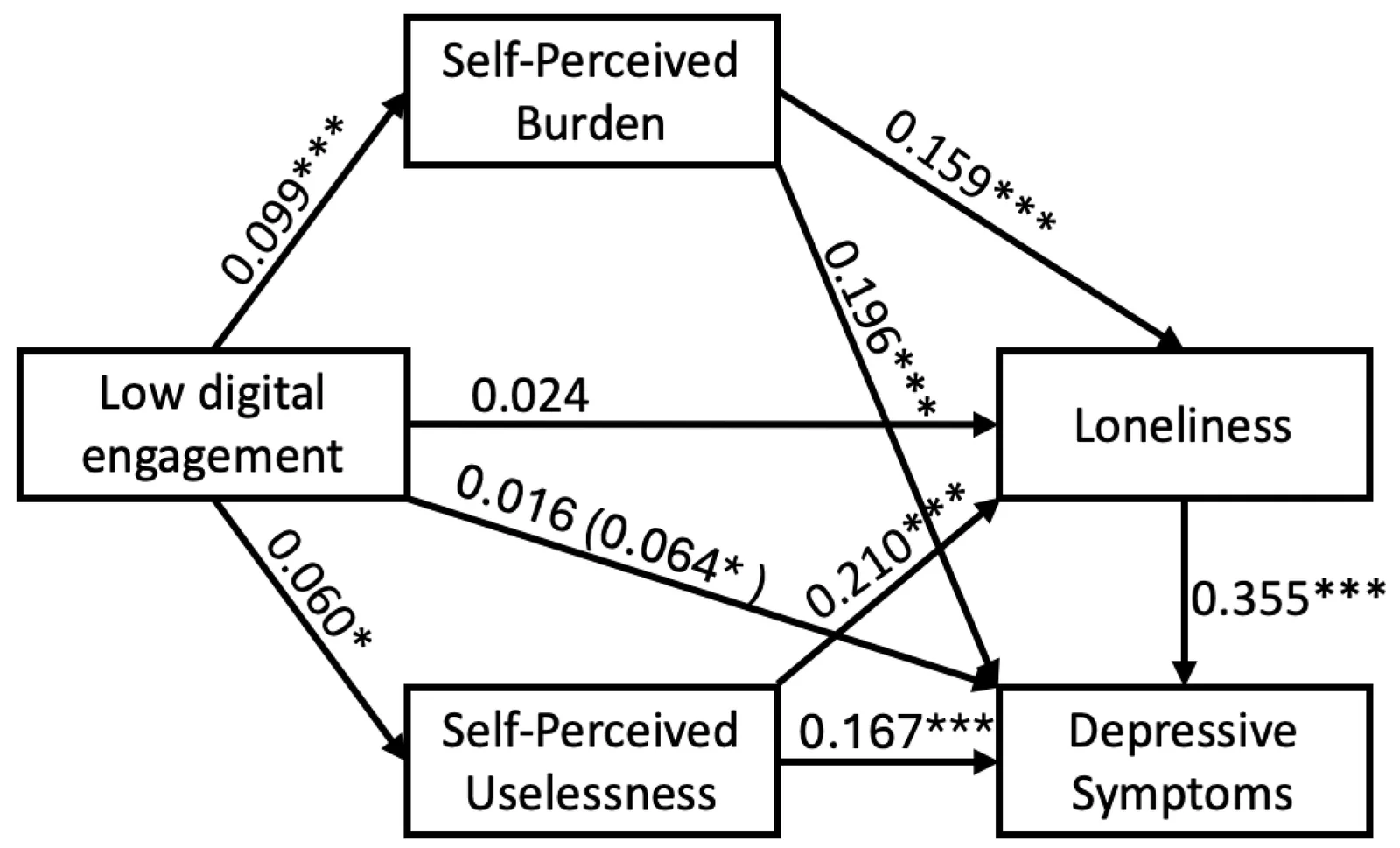
Path model of the association between low digital engagement and depressive symptoms through self-perceived burden, self-perceived uselessness, and loneliness (*N* = 1703). Standardized coefficients (β) are shown. All equations adjust for the full covariate set. * *p* < 0.05, *** *p* < 0.001.

**Figure 3:**
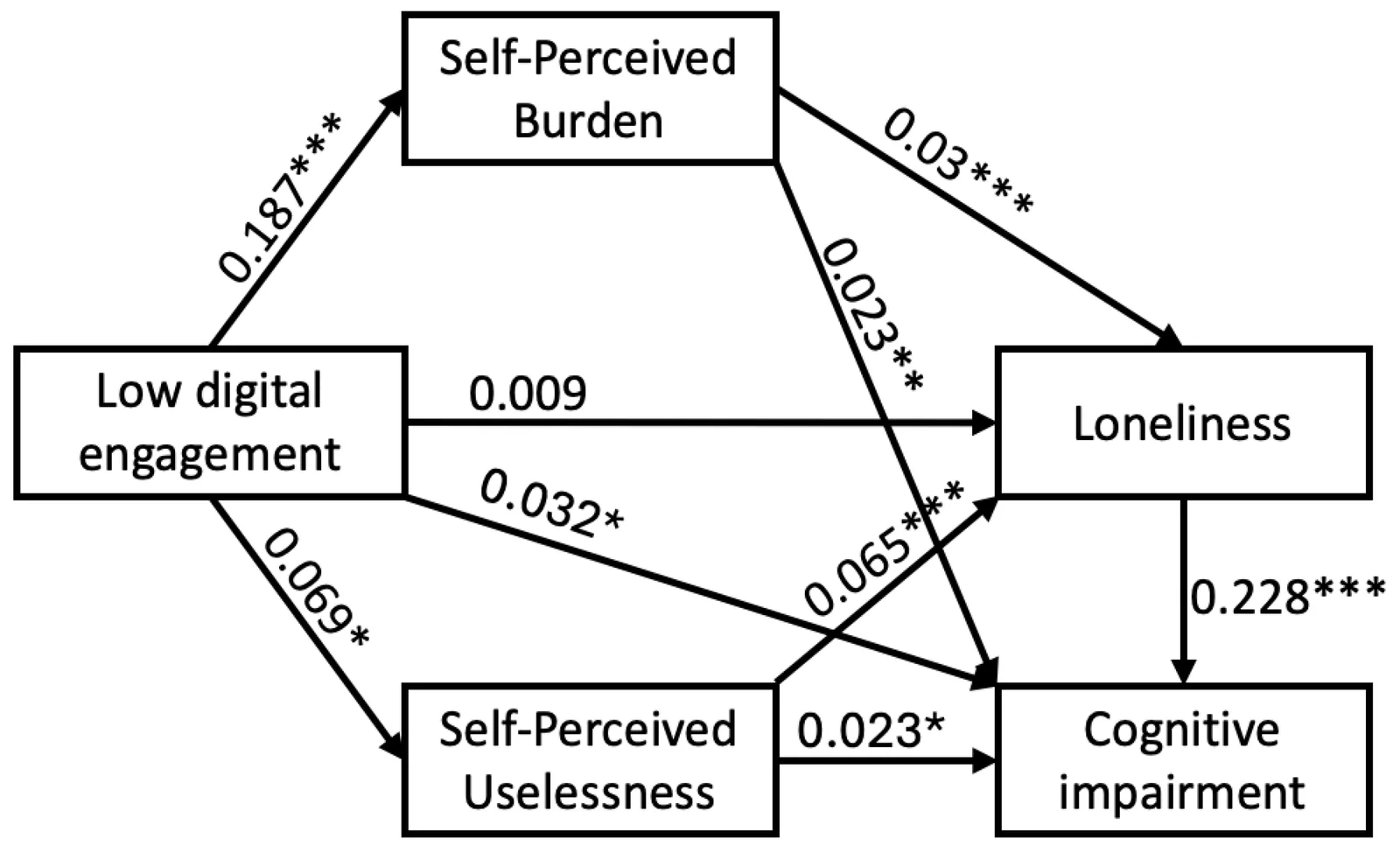
Path model of the association between low digital engagement and probable cognitive impairment (AD8 ≥ 2; logit link) through self-perceived burden, self-perceived uselessness, and loneliness (*N* = 1703). Coefficients on paths into the outcome are on the log-odds scale, mediator equations are linear. All equations adjust for the full covariate set. * *p* < 0.05, ** *p* < 0.01, *** *p* < 0.001.

**Table 1:** Participant characteristics (*N* = 1703).

Characteristic	Unweighted, *n* (%) or Mean ± SD	Weighted, Estimated Population *n* (%) or Mean
Age, years	68.59 ± 6.65	66.11 ± 7.31
Gender		
Male	709 (41.6)	3,187,961 (49.1)
Female	994 (58.4)	3,299,565 (50.9)
Marital status		
Not partnered	290 (17.0)	1,006,357 (15.5)
Married/cohabiting	1413 (83.0)	5,481,169 (84.5)
Hukou status		
Urban	1193 (70.0)	4,744,490 (73.1)
Rural	510 (30.0)	1,743,036 (26.9)
Education		
<High school	877 (51.5)	3,497,432 (53.9)
High school or equivalent	579 (34.0)	1,978,510 (30.5)
>High school	247 (14.5)	1,011,584 (15.6)
Annual household income quartile		
Q1	425 (25.0)	1,764,364 (27.2)
Q2	442 (26.0)	1,481,855 (22.8)
Q3	475 (27.9)	1,754,034 (27.0)
Q4	361 (21.2)	1,487,273 (22.9)
BMI category		
Underweight (<18.5)	51 (3.0)	192,840 (3.0)
Normal (18.5–23.9)	827 (48.6)	3,198,202 (49.3)
Overweight (24.0–27.9)	622 (36.5)	2,312,487 (35.6)
Obese (≥28.0)	203 (11.9)	783,996 (12.1)
Current smoker	321 (18.9)	1,597,932 (24.6)
Frequent drinker	189 (11.1)	939,756 (14.5)
Number of chronic conditions (0–7)	1.44 ± 1.25	1.28 ± 1.18
Any ADL limitation (Barthel < 100)	171 (10.0)	569,147 (8.8)
Living alone	230 (13.5)	774,009 (11.9)
Relatives and friends in regular contact	20.05 ± 19.54	18.71 ± 18.37
Low digital engagement (5–25)	16.07 ± 4.78	15.52 ± 4.71
Self-perceived burden (14–70)	29.16 ± 9.03	28.45 ± 9.14
Self-perceived uselessness (8–32)	15.57 ± 5.49	15.50 ± 5.68
Loneliness (3–12)	3.72 ± 1.71	3.78 ± 1.76
Depressive symptoms, CES-D-10 (0–30)	5.56 ± 4.64	5.47 ± 4.63
AD8 score (0–8)	1.73 ± 2.00	1.60 ± 1.93
Cognitive impairment (AD8 ≥ 2)	727 (42.7)	2,565,407 (39.5)

Notes: Weighted estimates use post-stratification weights calculated by Shanghai’s age-sex distribution; weighted frequencies are age-sex post-stratified estimated counts among approximately 6.49 million residents aged 55 to 85 years. For continuous variables, weighted estimates are survey-weighted means ± survey-weighted standard deviations. ADL = activities of daily living; BMI = body mass index; AD8 ≥ 2 denotes probable cognitive impairment.

**Table 2:** Path coefficients from the two path models linking low digital engagement, self-perceived burden, self-perceived uselessness, loneliness, and each outcome (*N* = 1703; 5000 bootstrap replications).

Path	b (95% CI)	Standardized β (95% CI)	*p*
*Outcome: depressive symptoms (linear SEM)*			
Self-perceived burden ← LDE (a1)	0.187 *** (0.093, 0.281)	0.099 *** (0.049, 0.149)	<0.001
Self-perceived uselessness ← LDE (a2)	0.069 * (0.007, 0.131)	0.060 * (0.006, 0.114)	0.030
Loneliness ← Burden (d1)	0.030 *** (0.019, 0.041)	0.159 *** (0.103, 0.216)	<0.001
Loneliness ← Uselessness (d2)	0.065 *** (0.049, 0.082)	0.210 *** (0.161, 0.259)	<0.001
Loneliness ← LDE (a3)	0.009 (−0.011, 0.029)	0.024 (−0.032, 0.081)	0.395
Depressive symptoms ← Burden (b1)	0.101 *** (0.076, 0.125)	0.196 *** (0.147, 0.245)	<0.001
Depressive symptoms ← Uselessness (b2)	0.141 *** (0.101, 0.180)	0.167 *** (0.121, 0.212)	<0.001
Depressive symptoms ← Loneliness (b3)	0.963 *** (0.821, 1.106)	0.355 *** (0.302, 0.407)	<0.001
Depressive symptoms ← LDE (c′)	0.015 (−0.027, 0.058)	0.016 (−0.028, 0.060)	0.480
**Path**	**b (95% CI)**	**Odds Ratio (95% CI)**	***p***
*Outcome: AD8 ≥ 2 (logit GSEM)*			
Self-perceived burden ← LDE (a1)	0.187 *** (0.093, 0.281)	—	<0.001
Self-perceived uselessness ← LDE (a2)	0.069 * (0.007, 0.131)	—	0.03
Loneliness ← Burden (d1)	0.030 *** (0.019, 0.041)	—	<0.001
Loneliness ← Uselessness (d2)	0.065 *** (0.049, 0.082)	—	<0.001
Loneliness ← LDE (a3)	0.009 (−0.011, 0.029)	—	0.395
AD8 ≥ 2 ← LDE (c′) †	0.032 * (0.004, 0.059)	1.032 (1.004, 1.061)	0.023
AD8 ≥ 2 ← Burden (b1) †	0.023 ** (0.009, 0.037)	1.023 (1.009, 1.038)	0.001
AD8 ≥ 2 ← Uselessness (b2) †	0.023 * (0.001, 0.046)	1.023 (1.001, 1.047)	0.044
AD8 ≥ 2 ← Loneliness (b3) †	0.228 *** (0.152, 0.304)	1.256 (1.164, 1.355)	<0.001

Notes: * *p* < 0.05; ** *p* < 0.01; *** *p* < 0.001 (two-tailed). LDE = low digital engagement. † Coefficients in the AD8 equation are on the log-odds scale; standardized coefficients are reported for the linear model only. A residual covariance between burden and uselessness was estimated in both models. All equations adjust for age, sex, marital status, hukou, education, household income quartile, BMI category, current smoking, frequent drinking, number of chronic conditions, ADL limitation, living alone, and number of relatives and friends in regular contact.

**Table 3:** Decomposition of the total associations of low digital engagement with depressive symptoms and probable cognitive impairment (*N* = 1703).

Component	b (95% CI)	Standardized β (95% CI)	% of Total
*Outcome: depressive symptoms*			
Total association (c)	0.062 * (0.011, 0.113)	0.064 * (0.012, 0.116)	100
Direct association (c′)	0.015 (−0.027, 0.058)	0.016 (−0.028, 0.060)	24.8
Total indirect association	0.047 ** (0.018, 0.075)	0.048 ** (0.019, 0.077)	75.2
LDE → Burden → Y	0.019 ** (0.008, 0.030)	0.019 ** (0.008, 0.030)	30.3
LDE → Uselessness → Y	0.010 * (0.001, 0.019)	0.010 * (0.001, 0.019)	15.6
LDE → Loneliness → Y	0.008 (−0.011, 0.028)	0.009 (−0.011, 0.029)	13.6
LDE → Burden → Loneliness → Y	0.005 ** (0.002, 0.009)	0.006 ** (0.002, 0.009)	8.7
LDE → Uselessness → Loneliness → Y	0.004 * (0.000, 0.009)	0.004 * (0.000, 0.009)	7
Contrast: serial burden − serial uselessness	0.001 (−0.004, 0.006)	0.001 (−0.004, 0.006)	—
Contrast: all via burden − all via uselessness	0.010 (−0.005, 0.026)	0.010 (−0.006, 0.026)	—
**Component**	**b (95% CI), log-odds**		
*Outcome: AD8 ≥ 2 (components on the log-odds scale)*			
Direct association (c′)	0.0315 * (0.0043, 0.0588)	—	—
Total indirect association	0.0102 ** (0.0032, 0.0173)	—	—
LDE → Burden → Y	0.0043 * (0.0009, 0.0078)	—	—
LDE → Uselessness → Y	0.0016 (−0.0005, 0.0037)	—	—
LDE → Loneliness → Y	0.0020 (−0.0027, 0.0067)	—	—
LDE → Burden → Loneliness → Y	0.0013 ** (0.0004, 0.0022)	—	—
LDE → Uselessness → Loneliness → Y	0.0010 (−0.0000, 0.0021)	—	—
Contrast: serial burden − serial uselessness	0.0003 (−0.0008, 0.0013)	—	—
Contrast: all via burden − all via uselessness	0.0030 (−0.0016, 0.0075)	—	—

Notes: Estimates are from the models in Table 2 (5000 bootstrap replications; delta-method, normal-based confidence intervals). Percentages are point estimates of each component divided by the total association and are reported for the linear model only; for the logit model, components are on the log-odds scale and do not sum to a total association. Contrast rows test the difference between the burden and uselessness routes. * *p* < 0.05; ** *p* < 0.01 (two-tailed).

## Data Availability

The datasets used and/or analyzed during the current study are available from the corresponding author on reasonable request.

## Supplementary Materials

The following supporting information can be downloaded at: https://www.mdpi.com/article/10.3390/bs16101726/s1. Table S1: Means, standard deviations, and Pearson correlations among core study variables (*N* = 1703); Table S2: Multivariable regressions of mediators and outcomes on low digital engagement and covariates (*N* = 1703); Table S3: Indirect associations of low digital engagement with depressive symptoms and probable cognitive impairment through each mediator separately (*N* = 1703; 1000 bootstrap replications); Table S4: Alternative orderings and reverse models (*N* = 1703; 1000 bootstrap replications); Table S5: Model fit and likelihood-ratio tests of the constrained models fixing the direct path LDE → loneliness to zero (*N* = 1703).
