# Supplementary material for: Low Digital Engagement, Depressive Symptoms, and Probable Cognitive Impairment Among Community-Dwelling Older Adults in Shanghai: A Cross-Sectional Path Analysis of Self-Perceived Burden, Uselessness, and Loneliness
Source: Behav Sci (Basel). 2026 Sep 23;16(10):1726. doi: 10.3390/bs16101726 (PMC13701919; doi:10.3390/bs16101726)
Supplement: Supplementary file 1 [file behavsci-16-01726-s001.zip › behavsci-4420881-supplementary.pdf]

Supplementary Table S1. Means, standard deviations, and Pearson correlations among core study variables (N = 1,703)

Variable	Mean (SD)	Low digital engagement	Self-perceived burden	Self-perceived uselessness	Loneliness	Depressive symptoms	AD8 score
Low digital engagement	16.07 (4.78)	1					
Self-perceived burden	29.16 (9.03)	0.221***	1				
Self-perceived uselessness	15.57 (5.49)	0.097***	0.399***	1			
Loneliness	3.72 (1.71)	0.124***	0.294***	0.295***	1		
Depressive symptoms	5.56 (4.64)	0.159***	0.437***	0.402***	0.498***	1	
AD8 score	1.73 (2.00)	0.236***	0.285***	0.249***	0.285***	0.410***	1

Notes: *p < .05; **p < .01; ***p < .001 (two-tailed). AD8 scores are the continuous 0–8 screening scores

Supplementary Table S2. Multivariable regressions of mediators and outcomes on low digital engagement and covariates (N = 1,703)

	Self-perceived burden	Self-perceived uselessness	Loneliness	Depressive symptoms	AD8 ≥ 2, Odds Ratio
Low digital engagement	0.187 (0.051)***	0.069 (0.032)*	0.019 (0.010)	0.062 (0.026)*	1.040 (0.014)**
Age, years	0.102 (0.034)**	−0.100 (0.022)***	−0.009 (0.007)	−0.034 (0.017)*	0.996 (0.009)
Male	−0.092 (0.532)	−0.630 (0.338)	−0.029 (0.103)	−0.668 (0.271)*	0.970 (0.131)
Married/cohabiting (ref: not partnered)	−0.515 (0.741)	−0.818 (0.471)	−0.974 (0.143)***	−1.030 (0.377)**	1.029 (0.194)
Urban hukou (ref: rural)	−0.525 (0.486)	0.462 (0.309)	0.004 (0.094)	−0.126 (0.247)	0.642 (0.078)***
Education level (per level)	0.364 (0.331)	−0.322 (0.210)	0.040 (0.064)	0.050 (0.168)	0.981 (0.083)
Household income quartile (per quartile)	−1.273 (0.234)***	−0.339 (0.148)*	−0.086 (0.045)	−0.317 (0.119)**	0.905 (0.054)
BMI (ref: normal)					
Underweight	0.972 (1.215)	0.298 (0.771)	0.246 (0.235)	1.275 (0.618)*	1.313 (0.402)
Overweight	−0.612 (0.446)	−0.067 (0.283)	0.056 (0.086)	−0.197 (0.227)	1.048 (0.120)
Obese	0.500 (0.658)	−0.029 (0.418)	−0.197 (0.127)	−0.259 (0.335)	0.934 (0.156)
Current smoker	−0.143 (0.635)	−0.465 (0.403)	−0.152 (0.123)	−0.226 (0.323)	0.610 (0.102)**
Frequent drinker	−0.950 (0.719)	−0.397 (0.457)	0.108 (0.139)	−0.256 (0.366)	0.841 (0.162)
Number of chronic conditions	1.250 (0.170)***	0.641 (0.108)***	0.129 (0.033)***	0.888 (0.087)***	1.310 (0.058)***
Any ADL limitation	4.130 (0.692)***	1.517 (0.439)***	0.357 (0.134)**	2.734 (0.352)***	2.274 (0.409)***
Living alone	1.874 (0.801)*	−0.838 (0.509)	0.368 (0.155)*	0.349 (0.408)	1.139 (0.233)
Relatives and friends in regular contact	−0.025 (0.011)*	−0.009 (0.007)	−0.006 (0.002)**	−0.016 (0.005)**	0.993 (0.003)*

Notes: Columns 1–4 are OLS regressions (unstandardized coefficients with standard errors); the AD8 ⩾ 2 column is a logistic regression reporting odds ratios with standard errors. Coefficients in bold indicate statistical significance. Education is modeled as ordinal level and income as quartile rank, matching the covariate specification of the path models. *p < .05; **p < .01; ***p < .001 (two-tailed).

Supplementary Table S3. Indirect associations of low digital engagement with depressive symptoms and probable cognitive impairment through each mediator separately (N = 1,703; 1,000 bootstrap replications)

	Loneliness		Self-perceived burden		Self-perceived uselessness	
	b	CI	b	CI	b	CI
Depressive symptoms	0.0236	(-0.0026, 0.0497)	0.0341***	(0.0163, 0.0518)	0.0198*	(0.0019, 0.0377)
Cognitive impairment (log-odds)	0.0052	(-0.0009, 0.0113)	0.0070**	(0.0028, 0.0112)	0.0037*	(0.0001, 0.0072)

Notes: *p < .05; **p < .01; ***p < .001 (two-tailed).

Supplementary Table S4. Alternative orderings and reverse models (N = 1,703; 1,000 bootstrap replications)

Model / outcome	Path	b (95% CI)
Depressive symptoms	LDE → Loneliness → Burden → Y	0.0023 (-0.0003, 0.0049)
Depressive symptoms	LDE → Loneliness → Uselessness → Y	0.0024 (-0.0005, 0.0052)
Depressive symptoms	LDE → Loneliness → Y	0.0182 (-0.0022, 0.0385)
AD8 ≥ 2 (log-odds)	LDE → Loneliness → Burden → Y	0.0005 (-0.0001, 0.0012)
AD8 ≥ 2 (log-odds)	LDE → Loneliness → Uselessness → Y	0.0004 (-0.0002, 0.0010)
AD8 ≥ 2 (log-odds)	LDE → Loneliness → Y	0.0043 (-0.0009, 0.0095)
Depressive symptoms (reverse)	Y → LDE (direct)	0.0190 (-0.0351, 0.0731)
Depressive symptoms (reverse)	Y → Loneliness → Burden → LDE	0.0030* (0.0005, 0.0056)
Depressive symptoms (reverse)	Y → Loneliness → Uselessness → LDE	0.0008 (-0.0026, 0.0042)
AD8 ≥ 2 (reverse)	Y → LDE (direct)	0.4971* (0.0871, 0.9072)
AD8 ≥ 2 (reverse)	Y → Loneliness → Burden → LDE	0.0254** (0.0065, 0.0444)
AD8 ≥ 2 (reverse)	Y → Loneliness → Uselessness → LDE	0.0057 (-0.0170, 0.0283)

Notes: *p < .05; **p < .01; ***p < .001 (two-tailed). All models are saturated and fit the data identically; the specified ordering cannot be adjudicated with cross-sectional data.

Supplementary Table S5. Model fit and likelihood-ratio tests of the constrained models fixing the direct path LDE → loneliness to zero (N = 1,703)

	Free model (all paths estimated)	Constrained model (LDE → loneliness = 0)
Panel A. Depressive symptoms (linear SEM; maximum likelihood)		
Log-likelihood	−48,549.92	−48,550.36
Number of parameters	78	77
AIC	97,255.85	97,254.72
BIC	97,680.18	97,673.61
Likelihood-ratio test vs. free model	—	$\chi^2(1) = 0.87$, p = 0.352
Fit indices of the constrained model		
χ^2 (model vs. saturated), df = 1		0.87, p = 0.352
RMSEA (90% CI); p-close		<0.001 (0.000, 0.062); 0.872
CFI		1
TLI		1.005
SRMR		0.001
Coefficient of determination (CD)		0.318
Panel B. Probable cognitive impairment, AD8 ≥ 2 (logit GSEM; maximum likelihood)		
Log-likelihood	−15,311.07	−15,311.50
Number of parameters	77	76
AIC	30,776.13	30,775.00
BIC	31,195.02	31,188.45
Likelihood-ratio test vs. free model	—	$\chi^2(1) = 0.87$, p = 0.352

Notes: Maximum likelihood estimates without bootstrap. In both models the constraint is not rejected, and the constrained model is favored by both AIC and BIC. In the free models the structural part is saturated (df = 0), so global fit indices are undefined. RMSEA, CFI, TLI, and SRMR are defined only for the linear SEM; they are not available for the logit specification, for which the likelihood-ratio test and information criteria serve as the fit comparison.
